# Impact of Multimorbidity on Disability and Quality of Life in the Spanish Older Population

**DOI:** 10.1371/journal.pone.0111498

**Published:** 2014-11-06

**Authors:** Noe Garin, Beatriz Olaya, Maria Victoria Moneta, Marta Miret, Antonio Lobo, Jose Luis Ayuso-Mateos, Josep Maria Haro

**Affiliations:** 1 Research Unit, Parc Sanitari Sant Joan de Déu, Universitat de Barcelona, Sant Boi de Llobregat, Barcelona, Spain; 2 Fundació Sant Joan de Déu, Esplugues de Llobregat, Barcelona, Spain; 3 Instituto de Salud Carlos III, Centro de Investigación Biomédica en Red de Salud Mental, CIBERSAM, Madrid, Spain; 4 Department of Psychiatry, Universidad Autónoma de Madrid, Madrid, Spain; 5 Department of Psychiatry, Hospital Universitario de La Princesa, Instituto de Investigación Sanitaria Princesa (IP), Madrid, Spain; 6 Department of Psychiatry, Universidad de Zaragoza and Instituto de Investigación Sanitaria de Aragón, Zaragoza, Spain; 7 Department of Psychiatry, Universidad Autónoma de Madrid, Madrid, Spain; San Francisco, United States of America

## Abstract

**Background:**

Population aging is closely related to high prevalence of chronic conditions in developed countries. In this context, health care policies aim to increase life span cost-effectively while maintaining quality of life and functional ability. There is still, however, a need for further understanding of how chronic conditions affect these health aspects. The aim of this paper is to assess the individual and combined impact of chronic physical and mental conditions on quality of life and disability in Spain, and secondly to show gender trends.

**Methods:**

Cross-sectional data were collected from the COURAGE study. A total of 3,625 participants over 50 years old from Spain were included. Crude and adjusted multiple linear regressions were conducted to detect associations between individual chronic conditions and disability, and between chronic conditions and quality of life. Separate models were used to assess the influence of the number of diseases on the same variables. Additional analogous regressions were performed for males and females.

**Results:**

All chronic conditions except hypertension were statistically associated with poor results in quality of life and disability. Depression, anxiety and stroke were found to have the greatest impact on outcomes. The number of chronic conditions was associated with substantially lower quality of life [β for 4+ diseases: −18.10 (−20.95,−15.25)] and greater disability [β for 4+ diseases: 27.64 (24.99,30.29]. In general, women suffered from higher rates of multimorbidity and poorer results in quality of life and disability.

**Conclusions:**

Chronic conditions impact greatly on quality of life and disability in the older Spanish population, especially when co-occurring diseases are added. Multimorbidity considerations should be a priority in the development of future health policies focused on quality of life and disability. Further studies would benefit from an expanded selection of diseases. Policies should also deal with gender idiosyncrasy in certain cases.

## Background

Population aging has gradually increased over the last years, with projections suggesting a two-fold increase in the worldwide population over 60 years old between 2013 and 2050 [1]. By then, the proportion of older people is expected to be double that of children in developed countries [2]. This demographic trend has led the European Commission to identify population aging as a crucial challenge in the 21^st^ century [3].

The aging process is associated with the onset of chronic conditions so that two thirds of elderly citizens in Europe suffer from multimorbidity, defined as the presence of at least two co-occurring conditions [4], [5]. Poor clinical and financial outcomes have been observed in patients with multimorbidity [6]. Chronic, non-communicable diseases are the biggest cause of death in high-income countries; responsible for more than 70% of deaths in 2008 [7]. Costs associated with chronic conditions have been estimated at 75% of total health expenditure, which is related to a wide range of health services such as hospitalization, medication, physician consultation, transportation, rehabilitation or long-term care [8], [9]. Health care in this context should aim to increase life span in a cost-efficient way while maintaining quality of life and the abilities required to perform daily-life activities [10].

Disability and quality of life are health outcomes which reflect the global health of the individual at various levels [11]–[14]. Disability is an umbrella term that reflects problems in bodily function, task performance and participation in life situations [12]. Quality of life is a broad multidimensional concept that includes both positive and negative aspects of life, and constitutes a major issue in the elderly [13], [14]. When analyzing the impact of chronic conditions on disability and quality of life, most studies have focused on the study of a single condition [15]. Lower quality of life and higher rates of disability have been found in people with chronic diseases such as arthritis, diabetes or asthma whilst limited or controversial results have been found for other conditions [16]–[21]. In other cases, the effect of chronic conditions on quality of life or disability has been assessed by using an index condition as a reference and the effects when considering the combination with other conditions [22]–[24]. This implies, for example, assessing how a specific index disease, such as diabetes, interacts with other conditions, e.g., diabetes and hypertension, diabetes and asthma. Comorbidity, or the study of these specific pairs of conditions, was introduced by Feinstein and adds very valuable information but also has its limitations [25]. Using this approach, an additive, synergistic or subtractive effect of these pair combinations can be explored. However, by using comorbidity pairs, neither the majority of all possible combinations nor the cumulative effects are studied, so that another, more comprehensive approach including the most relevant combinations is needed.

Multimorbidity is a relatively new concept that considers the co-occurrence of diseases in individual patients. This concept goes beyond the comorbidity definition, is not based on a central disease, and allows the assessment of the cumulative effect of chronic conditions [5]. Some recent studies have introduced comprehensive analyses including pairs, organ domains, or cumulative effects but there is still a need to deepen understanding of the additive impact of chronic conditions on disability and quality of life [26]–[29]. For example, McDaid et al (2013) recently presented a study assessing the effect of multiple chronic conditions on disability and quality of life in which the importance of co-occurring conditions was considered. One of its limitations was the classification of diseases into 4 groups (cardiovascular diseases, lung diseases, chronic pain, diabetes), so that the real impact of each condition was not assessed independently. Despite this limitation, it is one of the few papers assessing the effect of the number of chronic conditions on disability and quality of life [26]. Griffith et al (2010) also showed interesting results regarding chronic conditions taking multimorbidity into account. However, they opted to add pairs or triads of chronic conditions to the model rather than the number of chronic conditions [29].

Over recent years, the need to provide guidelines that consider the impact of co-occurring chronic conditions has been highlighted [25]. Thus, evaluation of the impact of multiple chronic conditions on quality of life and disability is essential as a first step to guide research and adjust guidelines, especially in Spain, where very little information is available [30], [31]. The importance of detecting the leading preventable causes of negative health outcomes, such as disability, is central to facilitating responses at a public health level. Thus, quantifying the chronic disease burden is essential [32]. Moreover, variability in methodology in previous studies underlines the need to provide results using standardized tools that allow cross-national comparisons in the future. Furthermore, gender differences are known to exist with respect to disability, quality of life and chronic diseases but very little is known about the relationship between them [18], [33], [34]. Finally, since most of the studies referring to index conditions have been carried out in a clinical setting, a more comprehensive public health perspective is needed focusing especially on the elderly, the age group most frequently affected by multimorbidity.

Understanding the factors that interact with disability and quality of life is essential to find ways of assessing, preventing and dealing with these issues at a public health level. This paper aims to assess the individual, combined and cumulative impact of chronic physical and mental conditions on quality of life and disability in a representative sample of older adults in the Spanish general population. Secondly, the paper aims to provide evidence on these issues across gender.

## Methods

### Design

The COURAGE in Europe project is a cross-sectional household survey conducted on a representative sample of the non-institutionalized adult population in Finland, Poland and Spain [35]. Data from the Spanish sample is analyzed in the current paper.

### Sample and procedures

A national representative sample of the Spanish adult population was selected by a stratified multistage clustered area probability method. A community-residing population over 18 years old was the target group. Three samples were chosen according to age: 18–49 years; 50–79 years; ≥80 years. Subgroups 50+ and 80+ years were oversampled, since these were the main target of the study. Exclusion criteria consisted of: lack of fluency in Spanish, house vacant/occupants elsewhere, deceased informant, individual not accessible [institutionalized/incarcerated/hospitalized), and the mentally unfit. Eligibility criteria were not met for 2,649 adults, with “occupants elsewhere/vacant house” the most common cause (74%). The survey protocol was translated from English into Spanish following WHO translation guidelines for assessment instruments [36]. Lay interviewers were trained on the survey before its administration. Quality assurance strategies were implemented during fieldwork [37]. The final response rate was 69.9%, corresponding to 4,583 adults over 18 years old. The response rate took into account the following issues: completed interview, partial interview, final refusal, inability to locate household or individual respondent, unsafe or dangerous area preventing the access to the interviewee and completed interviews not approved because of quality control problems. Of these, final refusal was the main cause of exclusion (80% of the overall excluded). Face-to-face structured interviews were conducted through Computer-Assisted Personal Interviewing (CAPI) at respondents' homes between July, 2011 and May, 2012. The interviewer judged whether the interviewee had cognitive problems at the beginning of the interview. This was a subjective judgment, which was indicated in case of clear memory problems or severe mental disorders. If unsure, the interviewer could ask two questions to help with the decision: “*a) How would you best describe your memory at present? Is it very good, good, moderate, bad or very bad? b) Compared to 12 months ago, would you say your memory is now better, the same or worse than it was then?*”. At this stage, respondents who answer “bad” or “very bad” to the first question and/or “worse” to the second question were used to consider the respondent had memory problems. Moreover, all proxy interviewees were evaluated by a supervisor to confirm this decision. In this case, a short version of the survey was administered to a proxy respondent. Data from proxy respondents was not analyzed because diagnosis of physical conditions and mental disorders was not performed in the proxy interviews. Thus, the final analysis consisted of 3,625 participants over 50 years old, once the 166 proxy respondents and the 792 participants younger than 50 years old had been removed.

### Data collection

Sociodemographic information included gender, age, marital status, education level, employment status and urbanicity. With regard to chronic physical conditions, participants were asked about having received a life-time diagnosis and treatment within the previous 12 months for angina, arthritis, asthma, chronic obstructive pulmonary disease (COPD), diabetes, edentulism, hypertension and stroke. Treatment was assessed with the following question: “Did you receive medication or other treatment for this disease during the last 12 months?”. Additionally, validated algorithms based on clinical symptoms were implemented to detect undiagnosed cases [38]. These algorithms come originally from the WHO's SAGE study and are in line with current clinical guidelines and reference publications [38]–[46]. When at least one of the two previous criteria was met, the respondent was considered to have one of the following conditions: arthritis, asthma, angina, stroke or chronic lung disease. Current cataract was assessed through self-reported medical diagnosis with co-occurring symptoms of cataract (visual problems associated with light sources and symptoms of blurred vision) to prevent the inclusion of respondents that had received corrective surgery. The symptoms were assessed, as in the WHO's SAGE study questionnaire, with the following questions: “*In the last 12 months have you experienced any of the following: a)… cloudy or blurry vision? b)… vision problems with light, such as glare from bright lights, or halos around lights?*” Hypertension, diabetes and edentulism did not have symptomatic algorithms since they are mostly asymptomatic conditions. Previous 12-months depression and anxiety were assessed with an adapted version of the World Health Organization Composite International Diagnostic Interview (CIDI), according to DSM-IV criteria [47]. For the assessment of functioning and disability we used the 12-item, validated version of the World Health Organization Disability Assessment Schedule 2.0 (WHODAS 2.0) [48]. Results range from 0 (no disability) to 100 (maximum disability). Quality of life was assessed through a modified version of the World Health Organization Quality of Life instrument (WHOQOL) called WHOQOL-AGE that has been specially adapted for the elderly population. This short-version contains 13 out of 100 questions from the original version and has been validated in populations over 50 years old [49]. Results range from 0 (minimum quality of life) to 100 (maximum quality of life).

### Statistical analysis

Unweighted frequencies, weighted proportions, means, confidence intervals and cross tabulations were applied for descriptive analysis. The Chi-square test was used to measure differences in prevalence of chronic diseases, number of conditions and sociodemographic variables across gender. T-test was used to assess differences in age, quality of life and disability across gender. T-test was also applied to evaluate differences in disability and quality of life for chronic conditions across gender.

To assess which pairs of conditions should be included in the analysis we took into account all 55 possible combinations from our 11 conditions. Frequency of disease pairs was computed. For those combinations with a co-occurrence higher than 1%, we calculated the multimorbidity coefficient as follows: real prevalence of the combination divided by the expected prevalence (expected prevalence  =  prevalence disease A x prevalence disease B) [50], [51]. We then selected the 10 pairs with the highest coefficient score, obtaining a list with the most prevalent and comorbid pairs in our population. The use of the multimorbidity index to further restrict our selection was made according to recent evidence in the study of comorbidity and multimorbidity that highlights the underlying structure (shared risk factors risks and biological links) present in some of these associations, which have great interest at several levels (understanding of ethiopathology, disease prevention, disease management, healthcare costs) [46], [52]. We fitted a linear regression model for every combination to test whether interactions were present with regard to the dependent variable: quality of life and disability. Those pairs with *p* value <0.2 were considered to interact and were selected for ulterior analysis.

Adjusted multiple linear regression was used to examine the association between physical conditions and quality of life in participants over 50 years old (n = 3,625). The model was adjusted for age, gender, educational level, marital status, urbanicity, all chronic conditions and those interactions with *p*<0.2: asthma with chronic obstructive lung disease (asthmaxCOPD); cataract with diabetes (cataractxdiabetes). This model was replicated using the number of chronic conditions rather than the individual variables to assess the additive effect in quality of life. Analogous procedures were applied to assess the association between chronic conditions and disability, considering the significant interactions for this outcome: depression with anxiety (depressionxanxiety) and cataract with diabetes (cataractxdiabetes).

Analogously, separate regressions were carried out for males and females to assess trends according to gender, as shown in the tables. To clarify the interpretation of these separate regressions by gender, additional statistical tests were performed: interactions between each chronic condition variable and gender were computed. Interactions were found in depression with respect to quality of life; and in diabetes, co-occurring cataract-diabetes, and the number of chronic conditions with respect to disability. The distinct impacts by gender have to be considered when interpreting the results of these diseases across gender.

Although beta-coefficients in the regressions can be considered a measure of effect size, we also computed the squared eta values with the aim of clarifying the meaning of our results. Squared eta is interpreted as the proportion of variance in the outcome explained by the variance in the independent variable. General recommendations for interpretation of squared eta results for ANOVA and GLM were followed: 0.01 small; 0.06 medium; 0.14 large. Depression, arthritis and the number of chronic conditions were considered to have large effect on outcomes. The other chronic condition variables produced moderate or small/moderate effect size values. Gender was considered to have moderate/small size effect.

The statistical analyses considered the complex nature of the sample design. Weights were used in analyses to adjust for differential probabilities of selection within households, and post-stratification weights to match the samples to socio-demographic distributions. Analyses were performed with IBM SPSS statistics 19.

### Ethics statement

The COURAGE study was approved by the Ethics Review Committee at Fundació Sant Joan de Déu, Barcelona, Spain and the Ethics Review Committee, La Princesa University Hospital, Madrid, Spain. Written informed consent was obtained from all participants. All investigators worked according to the principles expressed in the Declaration of Helsinki.

## Results

### Characteristics of participants

The study population consisted of 3,625 participants. A summary of sociodemographic data, chronic condition prevalence, disability results and quality of life score can be seen in Table 1. Prevalence of chronic conditions differs across gender except for hypertension, diabetes, asthma, stroke, edentulism and two of the co-occurring combinations assessed: asthma-COPD and cataract-diabetes. Women had higher rates of arthritis, depression, anxiety, cataracts and the combination depression-anxiety while men had higher prevalence of angina and COPD. 67.9% of the sample had at least one chronic condition. Gender differences were found with regard to the number of chronic conditions, with women having a greater number of chronic conditions (*p*<0.001). Women also had poorer results in quality of life and disability than men.

**Table 1:** Description of the sample of the Spanish Cohort of the COURAGE study.

	TOTAL	MALE	FEMALE	p value
Sample (n; %)	3625 (100%)	1643 (46.2%)	1982 (53.8%)	-
Age (mean, SE)	66.45 (0.18)	65.77 (0.27)	67.05 (0.24)	0.001
Education (n; %)				<0.001
no education	1207 (32.6%)	499 (29.7%)	708 (35.0%)	
primary	1075 (31.2%)	477 (30.2%)	598 (32.2%)	
secondary	949 (25.5%)	449 (27.2%)	500 (24%)	
university	393 (10.7%)	218 (12.9%)	175 (8.8%)	
Household income (n; %)				<0.001
1^st^ quintil	686 (22.0%)	304 (20.8%)	382 (23.1%)	
2^nd^ quintil	694 (21.7%)	254 (17.7%)	440 (28.2%)	
3^rd^ quintil	715 (21.6%)	311 (20.9%)	404 (25.6%)	
4^th^ quintil	745 (21.7%)	376 (25.0%)	369 (18.8%)	
5^th^ quintil	428 (13.0%)	229 (15.6%)	199 (10.7%)	
Marital Status (n; %)				<0.001
single	310 (8.5%)	148 (9.0%)	162 (8.0%)	
married	2258 (62.1%)	1262 (77.5%)	996 (48.9%)	
divorced	266 (6.9%)	101 (5.4%)	165 (8.2%)	
widow	791 (22.5%)	132 (8.1%)	659 (34.9%)	
Urbanicity (n; %)				0.649
urban	3138 (83.8%)	1421 (84.3%)	1717 (83.4%)	
rural	487 (16.2%)	222 (15.7%)	265 (16.6%)	
Work (n; %)				<0.001
retired	1385 (41.3%)	884 (58.2%)	501 (26.9%)	
other	1269 (35.7%)	241 (14.9%)	1028 (53.5%)	
working	817 (23.0%)	435 (26.9%)	382 (19.6%)	
QoL score (mean, SE)	71.02 (0.36)	73.35 (0.39)	69.02 (0.46)	<0.001
Disability score (mean, SE)	13.18 (0.52)	9.30 (0.50)	16.51 (0.74)	<0.001
Hypertension (n; %)	1331 (37.3%)	568 (35.6%)	763 (38.9%)	0.051
Diabetes (n; %)	514 (14.0%)	255 (15.1%)	259 (13.1%)	0.078
Angina (n; %)	236 (6.6%)	128 (7.8%)	108 (5.7%)	0.047
Asthma (n; %)	231 (6.3%)	90 (5.4%)	141 (7.1%)	0.064
Arthritis (n; %)	982 (26.8%)	266 (15.8%)	716 (36.3%)	<0.001
COPD (n; %)	233 (6.7%)	124 (8.3%)	109 (5.4%)	0.002
Stroke (n; %)	132 (4.6%)	62 (5.0%)	70 (4.3%)	0.428
Depression (n; %)	434 (12.1%)	117 (6.7%)	317 (16.7%)	<0.001
Anxiety (n; %)	55 (1.4%)	13 (0.6%)	42 (2.0%)	<0.001
Cataracts (n; %)	215 (6.0%)	62 (4.6%)	153 (7.2%)	<0.001
Edentulism (n; %)	677 (18.8%)	295 (17.3%)	382 (20.2%)	0.052
Asthma_COPD (n; %)	103 (2.9%)	55 (3.3%)	48 (2.5%)	0.205
Depression_anxiety (n; %)	42 (1.1%)	9 (0.4%)	33 (1.6%)	<0.001
Caratact_diabetes (n; %)	65 (1.7%)	21 (1.3%)	44 (2.0%)	0.060
Num chronic conditions (n; %)				<0.001
0	1173 (32.1%)	594 (36.4%)	579 (28.3%)	
1	1074 (29.6%)	531 (31.3%)	543 (28.2%)	
2	688 (18.5%)	275 (16.3%)	413 (20.4%)	
3	372 (10.7%)	133 (9.1%)	239 (12.1%)	
4+	318 (9.1%)	110 (6.8%)	208 (11.0%)	

Unweighted frequencies, and weighted means and proportions are displayed. Chi-square test for 2xN tables and T-test were performed to compare across gender. NOTE: Household income was divided into 5 quintiles (the first indicating the lowest income). Education category ‘no education’ included those people that had never been to school or did not finish primary school. Marital status ‘married’ category included currently married or cohabiting. Employment ‘other’ category included training, homemakers, unemployed, voluntary work, health problems, caring for family, sick leave, no need to work, temporary time off and voluntary work. Anxiety included Generalized Anxiety Disorder and Panic Disorder. Abbreviations: SE, standard error.

### Impact of chronic conditions on quality of life and disability

A summary of the scores for quality of life and disability are presented for every condition and for the number of conditions in Table 2. Depression, anxiety and stroke are the conditions with the highest impact on quality of life and disability scores. Hypertension is the condition with the lowest impact on quality of life and disabitlity of the diseases assessed. The number of chronic conditions is related to worse quality of life and disability.

**Table 2:** Quality of life and disability scores in the sample, overall and by gender.

	Disability	Disability Male	Disability Female	p value	QoL	QoL male	QoL female	p value
**Hypertension**	17.8 (16.2–19.3)	12.3 (10.4–14.1)	22.1 (20.1–24.1)	<0.001	69.2 (68.1–70.2)	72.1 (70.8–73.5)	66.8 (65.4–68.3)	<0.001
**Diabetes**	21.4 (19.4–23.5)	15.2 (12.5–17.8)	27.7 (25.0–30.4)	<0.001	66.6 (65.1–68.0)	69.5 (67.3–71.7)	63.6 (61.8–65.5)	<0.001
**Angina**	28.4 (25.3–31.5)	21.1 (17.6–24.7)	36.9 (31.8–41.9)	<0.001	62.9 (60.9–65.0)	67.7 (65.0–70.3)	57.4 (53.8–61.0)	<0.001
**Asthma**	27.0 (24.1–30.0)	24.1 (19.8–28.4)	28.9 (25.3–32.6)	0.109	62.9 (61.0–64.8)	64.8 (62.4–67.1)	61.7 (59.0–64.5)	0.109
**Arthritis**	24.4 (22.5–26.4)	20.2 (17.7–22.8)	26.0 (23.4–28.6)	0.002	65.2 (63.9–66.4)	67.9 (65.8–70.0)	64.2 (62.6–65.7)	0.005
**COPD**	29.5 (25.6–33.3)	25.9 (21.1–30.8)	34.1 (29.4–38.8)	0.014	61.3 (58.5–64.2)	64.1 (60.5–67.8)	57.7 (54.3–61.1)	0.006
**Stroke**	30.5 (25.5–35.4)	27.0 (20.9–33.0)	34.0 (27.5–40.5)	0.044	60.9 (56.7–65.2)	65.2 (62.1–68.3)	56.6 (48.5–64.7)	0.065
**Depression**	33.0 (29.6–36.3)	30.0 (25.3–34.6)	34.0 (29.4–38.5)	0.257	54.6 (52.7–56.5)	53.6 (51.0–56.3)	54.9 (52.4–57.3)	0.509
**Anxiety**	33.2 (28.6–37.9)	29.8 (16.3–43.8)	34.2 (28.6–39.8)	0.578	50.7 (47.9–55.1)	57.4 (47.9–66.9)	48.9 (44.8–52.9)	0.075
**Cataracts**	29.7 (26.5–32.9)	23.4 (18.2–28.5)	33.2 (29.6–36.8)	0.002	62.4 (60.5–64.3)	65.4 (62.2–68.6)	60.8 (58.4–63.2)	0.031
**Edentulism**	22.1 (20.2–24.0)	16.4 (14.0–18.8)	26.3 (23.6–29.1)	<0.001	66.9 (65.1–68.8)	69.5 (67.7–71.3)	65.0 (62.1–67.9)	0.11
**Asthma-COPD**	32.9 (29.6–36.2)	30.7 (25.7–35.7)	35.4 (29.8–40.96)	0.250	60.1 (57.8–62.4)	62.3 (59.2–65.5)	57.6 (53.6–61.6)	0.078
**Depression-anxiety**	35.98 (30.8–41.2)	33.2 (18.1–48.3)	36.6 (31.0–42,3)	0.639	47.1 (43.3–51.0)	52.7 (42.6–62.8)	45.8 (41.5–50.1)	0.190
**Caratact-diabetes**	41.7 (36.7–46.8)	35.6 (26.0–45.3)	45.0 (39.6–50.5)	0.091	55.7 (52.5–59.0)	59.6 (52.4–66.9)	53.7 (50.5–56.8)	0.145
**Num chronic conditions**								
** 0**	3.6 (3.0–4.1)	2.8 (2.2–3.4)	4.5 (3.6–5.3)	0.001	76.5 (75.6–77.4)	77.1 (76.1–78.0)	75.9 (74.7–77.0)	0.038
** 1**	9.6 (8.5–10.7)	6.9 (6.1–7.8)	12.2 (9.9–14.4)	<0.001	72.6 (71.2–74.0)	74.6 (73.4–75.8)	70.7 (68.9–72.6)	<0.001
** 2**	16.9 (15.4–18.4)	13.0 (10.5–15.5)	19.6 (17.9–21.3)	<0.001	70.2 (68.8–71.5)	72.8 (71.0–74.6)	68.3 (66.6–70.1)	<0.001
** 3**	24.1 (22.0–26.2)	18.5 (15.1–21.9)	27.8 (24.9–30.6)	<0.001	64.3 (62.9–65.7)	66.0 (63.9–68.2)	63.2 (61.3–65.1)	0.061
** 4+**	38.2 (35.4–40.9)	34.0 (29.1–38.8)	40.4 (37.1–43.7)	0.033	56.2 (53.5–58.9)	58.8 (55.9–61.7)	54.8 (51.0–58.6)	0.104

Weighted means of quality of life and disability results for people with each condition are displayed. Results are presented for the overall sample and by gender groups. Results with 95% Confidence interval. Statistical significance from gender comparison is showed (T-test analysis). NOTE: Abbreviations: QoL, quality of life.

Impact of single chronic conditions and multiple chronic conditions on disability are presented in Tables 3 and 4. Analogous information is given for quality of life in Tables 5 and 6. At a global level, the linear regression showed that, individually, each chronic condition was related to poorer results in quality of life and higher rates of disability, except for hypertension, where no statistically significant difference was found (Tables 3 and 5). In the regressions, higher educational level and being married were also related with better outcomes in quality of life and disability.

**Table 3:** Impact of chronic conditions on disability.

	Disability global		Disability male		Disability female	
	B (95% CI)	p value	B (95% CI)	p value	B (95% CI)	p value
**Intercept**	−11.75 (−17.76,−5.74)	<0.001	−5.18 (−11.34,0.97)	0.098	−14.68 (−22.30,−7.05)	<0.001
**Sex (ref. male)**	2.92 (1.72,4.12)	<0.001	-	-	-	-
**Age (per each additional year)**	0.32 (0.24,0.41)	<0.001	0.22 (0.12,0.31)	<0.001	0.42 (0.31,0.53)	<0.001
**Marital status (ref: married)**						
** divorced/separated**	−0.55 (−2.70,1.59)	0.609	1.46 (−0.82,3.76)	0.207	−1.50 (−4.23,2.23)	0.28
** widow**	2.15 (0.60,3.70)	0.007	1.66 (−0.94,4.26)	0.208	1.36 (−0.69,3.41)	0.192
** single**	0.61 (−1.11,2.33)	0.486	−0.13 (−1.91,1.65)	0.886	0.83 (−2.14,3.81)	0.582
**Education level (ref: no studies)**						
** primary**	−3.89 (−5.46/−2.320)	<0.001	−3.85 (−5.64,−2.06)	<0.001	−3.81 (−6.24,−1.38)	0.002
** secondary**	−4.62 (−6.23,−3.02)	<0.001	−5.15 (−7.05,−3.25)	<0.001	−4.09 (−6.17,−2.00)	<0.001
** university**	−5.80 (−7.99,−3.62)	<0.001	−4.92 (−6.69,−3.16)	<0.001	−6.78 (−10.16,−3.40)	<0.001
**Urbanicity (ref: rural)**	−3.17 (−5.07,−1.27)	0.001	−2.61 (−5.04,−0.19)	0.034	−3.58 (−6.13,−1.03)	0.006
**Chronic conditions**						
** depression**	15.70 (13.62,17.77)	<0.001	16.14 (12.10,20.17)	<0.001	15.60 (13.14,18.06)	<0.001
** anxiety**	11.17 (2.49,19.86)	0.012	11.40 (−11.52,34.32)	0.327	11.94 (1.32,22.56)	0.028
** angina**	6.87 (4.52,9.22)	<0.001	6.01 (3.31,8.71)	0.034	7.62 (3.55,11.70)	<0.001
** asthma**	2.34 (0.28,4.40)	0.03	4.00 (0.17,7.83)	0.041	1.42 (−1.35,4.20)	0.311
** COPD**	8.63 (6.21,11.05)	<0.001	9.12 (5.44,12.80)	<0.001	8.32 (4.84,11.80)	<0.001
** cataract**	3.28 (0.37,6.19)	0.027	0.08 (−4.82,4.97)	0.975	4.60 (1.01,8.18)	0.012
** arthritis**	7.50 (5.95,9.06)	<0.001	7.41 (4.92,9.90)	<0.001	7.38 (5.42,9.34)	<0.001
** diabetes**	2.32 (0.56,4.08)	0.010	0.79 (−0.95,2.53)	0.373	4.25 (1.57,6.94)	0.002
** hypertension**	0.14 (−0.97,1.25)	0.802	−0.37 (−1.60,0.86)	0.554	0.170 (−1.65,1.99)	0.854
** edentulism**	2.93 (1.19,4.68)	0.001	3.08 (1.00,5.16)	0.004	2.79 (0.03,5.54)	0.048
** stroke**	12.15 (8.08,16.22)	<0.001	15.90 (9.66,22.14)	<0.001	9.17 (4.13,14.21)	<0.001
**Interactions**						
** depression-anxiety**	−6.54 (−15.96,2.89)	0.172	−2.59 (−28.70,23.52)	0.845	−8.04 (−19.52,3.43)	0.168
** caratact-diabetes**	9.76 (4.27,15.25)	0.001	15.41 (5.99,24.84)	0.002	6.28 (−0.59,13.15)	0.073

Linear regression model for the global sample was adjusted for sex, age, marital status, education level, urbanicity, individual chronic conditions and interactions. Analogous linear regressions were performed for male and female, adjusted by the same variables but sex. Results with 95% Confidence interval.

**Table 4:** Impact of multiple chronic conditions on disability.

Number of chronic conditions (ref: not having a condition)	Disability global		Disability male		Disability female	
	B (95% CI)	p value	B (95% CI)	p value	B (95% CI)	p value
**1**	3.57 (2.56,4.56)	<0.001	2.80 (1.66,4.85)	<0.001	4.40 (2.67,6.13)	<0.001
**2**	8.59 (6.92,10.27)	<0.001	7.32 (5.14,9.49)	<0.001	9.90 (7.54,12.26)	<0.001
**3**	14.61 (12.54,16.69)	<0.001	13.40 (10.06,16.74)	<0.001	15.59 (12.69,18.48)	<0.001
**4+**	27.64 (24.99,30.29)	<0.001	27.11 (2.38,22.40)	<0.001	28.23 (24.82,31.63)	<0.001

Linear regression model for the global sample was adjusted for sex, age, marital status, education level, urbanicity and number of chronic conditions. Analogous linear regressions were performed for male and female, adjusted by the same variables but sex. Results with 95% Confidence interval.

**Table 5:** Impact of chronic conditions on quality of life.

	QoL global		QoL male		QoL female	
	B (95% CI)	p value	B (95% CI)	p value	B (95% CI)	p value
**Intercept**	72.80 (69.41,76.19)	<0.001	72.89 (67.89,77.88)	<0.001	73,79 (68.65,78.93)	<0.001
**Sex (ref. male)**	−0.91 (−1.81,−0.01)	0.048	-	-	-	-
**Age (per each additional year)**	0.04 (−0.02,0.09)	0.166	0.05 (−0.02,0.11)	0.197	−0.01 (−0.08,0.07)	0,918
**Marital status (ref: married)**						
** divorced,separated**	−6.63 (−8.48,−4.77)	<0.001	−8.82 (−11.98,−5.67)	<0.001	−5.07 (−7.37,−2.76)	<0.001
** widow**	−2.73 (−4.05,−1.42)	<0.001	−3.39 (−5.62,−1.15)	0.003	−1.71 (−3.30,−0.12)	0.035
** single**	−3.47 (−5.43,−1.52)	0.001	−5.55 (−8.15,−2.95)	<0.001	−0.83 (−3.70,2.03)	0.566
**Education level (ref: no studies)**						
** primary**	2.76 (1.40,4.12)	<0.001	2.43 (0.55,4.30)	0.011	2.93 (1.16,4.70)	0.001
** secondary**	5.08 (3.32,6.82)	<0.001	4.48 (2.44,6.52)	<0.001	5.36 (3.01,7.72)	<0.001
** university**	7.75 (6.02,9.47)	<0.001	6.51 (4.27,8.75)	<0.001	8.34 (5.87,10.85)	<0.001
**Urbanicity (ref: rural)**	−0.96 (−2.70,0.79)	0.279	−0.78 (−2.77,1.22)	0.443	−1.49 (−3.65,0.673)	0.176
**Chronic conditions**						
** depression**	−14.00 (−15.85,−12.14)	<0.001	−17.00 (−19.98,−14.03)	<0.001	−12.79 (−15.15,−10.44)	<0.001
** anxiety**	−7.82 (−11.57,−4.08)	<0.001	−3.60 (−9.40,−2.19)	0.221	−9.29 (−13.77,−4.80)	<0.001
** angina**	−3.45 (−5.20,−1.69)	<0.001	−2.30 (−4.71,0.10)	0.060	−4.67 (−7.55,−1.78)	0.002
** asthma**	−3.40 (−5.49,−1.32)	0.002	−4.66 (−7.05,−2.26)	<0.001	−2.57 (−5.40,0.26)	0.075
** COPD**	−6.19 (−8.39,−4.00)	<0.001	−5.33 (−8.61,−2.05)	0.002	−7.00 (−10.51,−3.49)	<0.001
** cataract**	−2.49 (−4.61,−0.37)	0.022	−2.58 (−5.56,0.40)	0.089	−2.35 (−5.08,0.38)	0.091
** arthritis**	−3.52 (−4.65,−2.38)	<0.001	−3.52 (−5.31,−1.73)	<0.001	−3.45 (−4.83,−2.07)	<0.001
** diabetes**	−1.49 (−2.87,−0.11)	0.035	−1.60 (−3.29,0.10)	0.065	−1.21 (−3.24,0.82)	0.241
** hypertension**	0.38 (−0.50,1.26)	0.397	0.538 (−0.69,1.77)	0.389	0.45 (−0.88,1.77)	0.505
** edentulism**	−1.63 (−3.01,−0.26)	0.020	−2–21 (−3.85,−0.58)	0.008	−1.08 (−3.23,1.07)	0.322
** stroke**	−8.16 (−11.77,−4.55)	<0.001	−7.93 (−11.47,−4.38)	<0.001	−8.88 (−15.34,−2.42)	0.007
**Interactions**						
** asthma-COPD**	5.17 (1.50,8.85)	0.006	5.44 (0.41,10.48)	0.034	5.80 (−0.74,12.33)	0.082
** caratact-diabetes**	−4.01 (−8.06,0.05)	0.053	−4.64 (−13.10,3.83)	0.281	−3.87 (−8.71,0.97)	0.116

Linear regression model for the global sample was adjusted for sex, age, marital status, education level, urbanicity, individual chronic conditions and interactions. Analogous linear regressions were performed for male and female, adjusted by the same variables but sex. Results with 95% Confidence interval.

**Table 6:** Impact of multiple chronic conditions on quality of life.

Number of chronic conditions (ref: not having a condition)	QoL global		QoL male		QoL female	
	B (95% CI)	p value	B (95% CI)	p value	B (95% CI)	p value
**1**	−3.29 (−4.66,−1.93)	<0.001	−1.98 (−3.37,−0.60)	0.005	−4.68 (−6.73,−2.62)	<0.001
**2**	−5.15 (−6.62,−3.69)	<0.001	−3.89 (−5.74,−2.04)	<0.001	−6.15 (−8.15,−4.14)	<0.001
**3**	−10.67 (−12.34,−9.00)	<0.001	−11.01 (−13.42,−8.60)	<0.001	−10.70 (−12.93,−8.48)	<0.001
**4+**	−18.10 (−20.95,−15.25)	<0.001	−16.85 (−19.93,−13.77)	<0.001	−19.04 (−23.19,−14.88)	<0.001

Linear regression model for the global sample was adjusted for sex, age, marital status, education level, urbanicity and number of chronic conditions. Analogous linear regressions were performed for male and female, adjusted by the same variables but sex. NOTE: QoL  =  quality of life. Results with 95% Confidence interval.

Co-occurring diabetes and cataracts were found to be associated with higher disability (β: 9.76; 95% CI: 4.27, 15.25) and lower quality of life (β: −4.01; 95% CI: −8.06, 0.05) (table 3 and table 5).

Co-occurring asthma and COPD was associated with positive quality of life (β: 5.17; 95%CI: 1.50, 8.85). Co-occurring depression and anxiety showed lower disability but the result was not statistically significant (β: −6.54; 95CI: −15.96, 2.89).

Suffering from several chronic conditions was associated with higher disability, with scores in the questionnaire ranging from 3.6 (95% CI: 3.0, 4.1) to 38.2 (95% CI: 35.4, 40.9) when comparing people with no diseases and people with 1 and 4+ conditions respectively.

Similar changes were found with regard to the number of conditions and quality of life, where the score fell from 76.5 (95% CI: 75.6, 77.4) to 56.2 (95% CI: 53.5, 58.9). The regressions showed a strong association between the number of chronic conditions and both higher disability and worse quality of life (Table 4 and Table 6).

### Gender trends in quality of life and disability

As for diseases considered independently, women had poorer results in disability scores compared with men with the same conditions, except for asthma, depression and anxiety where no difference was found (Table 2). Similar results were found in quality of life although, in this case, stroke also showed no difference across gender (Table 2). At an additive level, women also had worse outcomes when considering a specific number of chronic conditions, although having 4+ chronic conditions was related to similar outcomes.

With regard to the regressions results, anxiety and angina were only related to worse quality of life in women, while asthma and edentulism were associated with worse results only in men (table 5). For disability, separate regressions for men and women also showed particular trends. Anxiety, cataract and diabetes were only associated with poorer results for women while asthma was associated with poorer results only in men (table 3). An increasing positive association between the number of conditions and poor results in disability and quality of life was found for both sexes (table 4, table 6). Being single resulted in poorer results in quality of life compared with being married only in men (table 5).

## Discussion

This study has shown that there is a strong association between chronic conditions and poor results in disability and quality of life, both at individual and additive level. Our study also showed relevant trends according to gender in this association.

### Individual associations

The most remarkable result is that mental disorders (depression and anxiety) have a higher impact on quality of life and disability than most chronic physical conditions. Some studies have suggested an intimate association between mental disorders and changes in quality of life and disability in the elderly but they tend to focus specifically on mental conditions or combinations of mental disorders so that individual qualitative or quantitative comparisons between different mental and physical chronic diseases are not available [27], [28], [53]–[55]. Moreover, a high proportion of studies to date did not include mental disorders when analyzing chronic conditions [15], [26], [29], [56]. There are few studies available which include mental and physical conditions, which show mixed results, so our results highlight the importance of mental health in the elderly at this level [10], [57]–[59]. Stroke is the physical condition with the highest impact on quality of life, followed by COPD, arthritis and angina. These conditions share some similarities such as physical limitations or disabling symptoms (pain, shortness of breath, etc.), having been previously associated with poor health outcomes [26], [60]–[65]. Asymptomatic conditions such as diabetes or edentulism resulted in a lower impact on quality of life. In this regard, our results complement the results of previous studies that found a mixed effect of diabetes on quality of life and disability but, in this study, we provide the additional context of other chronic conditions [31], [34], [56], [59], [66]–[68]. Hypertension turned out not to be associated with worse outcomes while earlier studies have shown mixed results [15], [19], [34], [56], [69]–[71]. When interpreting our results, it has to be considered that the hypertension diagnosis was reported by the respondents, so that a great proportion may have been receiving medical care at the time of the interview. It has been shown that symptoms are responsible for the greatest impact on quality of life in patients with hypertension so that patient monitoring and treatment would minimize the few symptoms present in the participants with hypertension [72]. Efforts to improve quality of life and disability should focus on prioritizing mental disorders and physical symptomatic conditions.

### Pair combinations

With regard to co-occurring pairs of conditions, suffering from diabetes and cataracts resulted in a synergic effect on disability and quality of life. These results must be treated with caution. Prevalence of cataracts is known to be strongly related to the duration of diabetes and parameters reflecting poor diabetes management, such as high levels of HbA1c, fasting blood sugar or macroalbuminuria [73], [74]. This poor control of diabetes, which could also be related to other metabolic syndrome complications, would lead the individual to a poorer health status and higher degree of disability compared with the expected addition of the individual effects of diabetes and cataracts.

Co-occurrence of asthma and COPD resulted in contrary directionality of the results compared with the individual effect of the diseases on quality of life. It does not alter the individual negative effect of asthma and COPD on quality of life but suggests a ceiling effect when having them simultaneously. Both asthma and COPD are highly prevalent conditions in the elderly and this has been defined as the asthma-COPD overlap syndrome, which describes a frequency of overlapping diagnoses over 50% in COPD patients aged over 80 years [75]. As the name suggests, this situation involves features of both conditions and has recently been related to poorer quality of life than that found in asthma cases and similar to that of COPD [76]. In our case, 43% of the participants diagnosed with COPD also suffered from asthma, which supports the results seen in clinical settings. Some clinical outcomes, which may differ from the individual conditions, could be related to these results. For example, Fu et al. (2013) found, in a longitudinal study, that patients with asthma-COPD overlap had a better prognosis than COPD or asthma patients, although other studies have showed more severe exacerbations when these conditions co-occur [77], [78]. Further research is needed to describe the asthma-COPD overlap and its impact on quality of life.

Co-occurring depression and anxiety resulted in lower effects on disability than theoretically expected although these results were not statistically significant. Further study is needed to clarify the combined impact of mental disorders in quality of life and disability due to the close relationship between these disorders.

### Additive impact of chronic conditions

At an additive level, there is a sharp and continuous decrease in quality of life when suffering from more chronic conditions, with Beta ranging from −3.26 (95% CI: −4.66, −1.93) in the group of respondents with one chronic disease to −18.10 (95% CI: −20.95, −15.25) in the group with four and more conditions, which underlines the relevance of multimorbidity in this outcome. Similar results are found when assessing the association between multimorbidity and disability with Beta results ranging from 3.57 (95% CI: 2.56, 4.56) in respondents with one condition up to 27.64 (95% CI: 24.99, 30.29) in patients with four and more conditions. These results expand and complement the evidence since most studies have focused on the impact of individual conditions, specific pairs of conditions or organ domain classifications. [10], [15], [22], [28], [29], [34], [53], [55], [57], [69], [70], [79], [80]. Our results support the descriptive analysis made by Lawson et al. (2013) in which participants reporting longstanding conditions presented reductions in preference-weighted health-related quality of life. Their results, however, are not completely comparable since they counted up to three chronic diseases, considered different conditions and the count itself only allowed one condition for every organ-based classification group [81]. Brettschneider et al. (2013) and Heyworth et al. (2009) found that overall quality of life and its dimensions, measured with the EQ-5D, decreased with an increasing number of chronic diseases [59,82). However, the study by Brettschneider et al. (2013) considered multimorbidity as a continuous variable without assessing the impact of the specific disease count, while Heyworth et al. (2009) only took six conditions into account, excluding mental health, so that results are complementary rather than comparable. On the other hand, Tan et al. (2013) also found poorer results in quality of life with a higher number of chronic conditions. However, there seemed to be a ceiling effect between two and three chronic conditions while, in our results, the group with four or more conditions shows a noticeably lower quality of life compared with respondents with three chronic conditions [71]. Our results in quality of life should help to target multimorbidity patients as population subgroups in which clinical, community and patient-centered care should be prioritized to ensure the best possible quality of life [83]. With regard to disability, little effort has previously been made in considering the additive effect of chronic conditions, as stated above. Our results suggest that multimorbidity patients require special attention due to the association between the number of chronic conditions and disability rates. Since disability per se predicts future disability status and is related to poor health outcomes, it is important to identify high-risk groups to develop preventive, curative or palliative strategies [34]. For example, patients at risk can benefit from interventions, such as resistance strength training or preventive home visitation programs [84].

### Gender trends

It is known that systematic gender-dependent errors can be made when analyzing the results of a study due to androcentrism or gender insensitivity [85]. Previous research on these topics has highlighted the specific need to address disparities and differences in risk and interventions across gender groups of people with chronic conditions [83]. Studies tend to consider gender when adjusting the regression models but only some of them provide descriptive results or separate analyses by gender, which in turn are usually focused on specific conditions [10], [29], [33], [63], [68], [86]–[88]. Consequently, our results covered the global sample as well as those for males and females.

Our results have shown that women had higher risk of disability than men after adjusting for covariates (β: 2.92; 95% CI: 1.72, 4.12) although the clinical relevance of this result is unclear since there are no clinical cut-offs for these types of screening tools (effect size resulted in moderate-small values). This result is comparable with the effect of diseases, such as diabetes (β: 2.32; 95% CI: 0.56, 4.08), but very low compared with depression (β: 15.70; 95% CI: 13.62, −17.77) or stroke (β: 12.15; 95% CI: 8.08, −16.22). Analogous results were found for quality of life.

Our results show higher disability and lower quality of life average scores in women than in men for most chronic conditions (e.g. women with angina had considerably higher scores for disability and lower for quality of life compared with men). These results reinforce the general idea that greater attention should be paid to women to prevent and manage poor outcomes in disability and quality of life. Additionally, it specifically clarifies this topic with regard to chronic conditions [68], [89]–[91]. However, similar quality of life and disability scores were found in both sexes for depression, anxiety, asthma and stroke (only for quality of life in stroke). When comparing the global scores across gender, a greater impact is seen in women but this difference disappears with respect to quality of life when three or more conditions are present. The impact of the increasing number of chronic conditions would appear to be similar across gender when people reach a certain level of multimorbidity.

When assessing the regression models for males and females, most conditions showed similar behavior as the reported in the regression including all participants. For some conditions, however, this association disappeared in men or women. Anxiety and angina were statistically related to poorer results in women only, while asthma and edentulism were related to poorer results solely in men. Since anxiety prevalence in men was relatively low, we think this result may be biased by the power of the study. Analogously, anxiety, cataracts and diabetes were associated with higher rates of disability solely in women while asthma was found to be related to higher disability in men. With regard to the number of chronic conditions, a greater impact on quality of life and disability was found in both genders. These results suggest that the effect of conditions in each gender group may differ and should be considered in future studies. With regard to asthma, for example, the management of the disease in men should focus particularly on preventing loss of quality of life and physical functioning.

### Strengths and limitations

Our study's main strength is that results are extrapolated to the entire Spanish older adult population. In the future, comparison will be possible with other countries included in the COURAGE and SAGE studies. It is also remarkable that the selection of chronic diseases, including depression and anxiety, were mainly omitted in previous studies despite having been related to poor health outcomes at an individual level. Diagnosis by means of both self-report plus symptom algorithms also allows a more complete picture of the participants to emerge compared with other studies. There are, however, limitations in our study. Its cross-sectional nature identifies associations but does not allow conclusions on cause-and-effect relationships to be drawn. Moreover, age effects may not be distinguished from cohort effects. Longitudinal studies are needed to better establish the association between multimorbidity, quality of life and disability, thus reducing this bias. Multimorbidity studies would benefit from a standardized definition and disease inclusion criteria [50]. For example, the exhaustive “Expanded Diagnosis Clusters of the ACG” system have been used in some studies, although it becomes complex to employ outside the clinical setting and in the case of poor integration of health care levels [92]. The choice of chronic conditions is also relevant since it is known that a higher number of assessed conditions results in a higher proportion of multimorbidity [5]. Our selection of chronic conditions was made according to the SAGE study, focusing on a limited number of highly prevalent conditions that are a major cause of disability, through a method that can be applied across countries. There is, however, a need to include diagnoses of other common conditions known to have a considerable impact on quality of life, disability and health care resources, in future studies. Research on multimorbidity, as highlighted by the recent review by Prados-Torres et et al. (2012), may include diseases such as malignancies, congestive heart failure or anemia [92].Moreover, when assessing specific pairs, we chose those highly prevalent pairs of conditions with a high degree of interaction shown in the multimorbidity index. However, further studies including other combinations are required to deepen knowledge of less common co-occurring conditions. The self-reported data-collection method could also bias the results, but this effect may be minimized as a good correlation between medical records and self-reported diagnosis has been found [93], [94]. Our analysis does not allow consideration of the progression and severity of conditions, which would be advisable in future studies. For example, severe COPD cases or poor glycemic control in diabetes may be related to poorer health outcomes [95]. There is a possibility that some respondents who did not take the medication prescribed by their doctors answered that they were not receiving treatment and were incorrectly classified as “not suffering from a specific disease”. This limitation is minimized since the question was quite open “did you received treatment”, rather than asking whether they were “taking the medication” and also due to the inclusion of the symptoms algorithm in most conditions. With regard to the analyses including the number of chronic conditions, further study is needed to clarify if greater contribution to the results is due to some conditions rather than others. We considered theoretical similar impact in our analyses to be consistent with the previous literature on that regard. Another limitation when analyzing the results is the geopolitical context. Specific results for an individual condition may vary according to external factors that should be analyzed if detected [96], [97]. Although financial crises may impact some results, such as the prevalence of mental disorders, recent evidence suggests that health in Spain has continued to improve during the first four years of the current economic recession, so it seems this bias would be reduced [98], [99]. Finally, separate results for women and men (average scores and regression models) obtained in our article have shed some light on gender issues but further efforts focusing on differences across gender would be needed in future studies.

## Conclusion

The results of this study contribute to a deeper understanding of the effect of chronic conditions on quality of life and disability. In Spain, multimorbidity is a prevalent phenomenon among elderly people in the community that increasingly affects both disability and quality of life as more co-occurring conditions accumulate. Multimorbidity patients should be considered as targets for clinical, community and patient-centered care based on preventive, curative or palliative strategies. Our results are especially relevant since little effort has been previously made to consider the additive effect of common chronic conditions on quality of life and disability. At an individual level, efforts to improve quality of life and disability should prioritize prevention and management of mental disorders and physical symptomatic conditions since they are associated with poorer outcomes than mainly asymptomatic conditions such as hypertension. Our results also highlight the need to include mental disorders, selected in very few previous studies, when analyzing multimorbidity because of their great impact on the results. Finally, there is need to consider gender as an important factor when assessing multimorbidity and designing interventions for multimorbidity patients since specific trends arise in some outcomes with women showing worse health results in most cases.
